# Evaluation of Oral Hygiene-Related Mobile Apps for Children in Sub-Saharan Africa

**DOI:** 10.3390/ijerph191912565

**Published:** 2022-10-01

**Authors:** Aida Kanoute, Florence Carrouel, Jocelyne Gare, Serigne Ndame Dieng, Amadou Dieng, Mbathio Diop, Daouda Faye, Laurie Fraticelli, Denis Bourgeois

**Affiliations:** 1 Public Health Service, Department of Dentistry, Cheikh Anta Diop University, Dakar 10700, Senegal; 2 Health, Systemic, Process (P2S), UR 4129 Research Unit, University Claude Bernard Lyon 1, University of Lyon, 69008 Lyon, France; 3 Public Health Laboratory (LASAP), ED2S Doctoral School of Sciences and Health, University Joseph Ki Zerbo, Ouagadougou 7021, Burkina Faso; 4 Hospices Civils de Lyon (HCL), 69002 Lyon, France

**Keywords:** oral health, education, health promotion, mobile app, prevention, child, toothbrushing

## Abstract

In sub-Saharan Africa, oral health is a real epidemiological challenge. Mobile applications represent a hope for the learning of oral hygiene in children and the fight against oral diseases. This study overviews and assesses the quality of mobile applications linked to oral hygiene for children currently featured on the iOS and Android stores in sub-Saharan Africa. Ten oral health professionals (OHP) used the French Mobile App Rating Scale (MARS-F) to rate 15 selected applications. The highest MARS-F scores for overall quality were reported for *Bonne nuit Caillou* (3.89 ± 0.74), *Mon Raccoon* (3.63 ± 0.95), and Chomper Chums (3.54 ± 0.54) while the lowest MARS-F scores for overall quality were achieved by Brushing time (2.31 ± 0.61), *De belles dents* (2.55 ± 0.55) and Brushing Hero (2.77 ± 0.53). The subjective quality scores ranged from 1.50 ± 0.68 for Brushing time to 3.25 ± 0.97 for *Bonne nuit Caillou*. Specificity scores ranged from 1.95 ± 0.88 (Brushing time) to 3.75 ± 0.84 (*Bonne nuit Caillou*). Thus, OHP rated positively the quality of the majority of mobile applications linked to oral hygiene for children, their effect on users’ knowledge, attitudes, and intentions to change, and the probability of effective oral hygiene behavior modification. They stated that they would recommend their use to their patients who need them. However, studies analyzing the change in oral hygiene behavior of children using these apps need to be conducted.

## 1. Introduction

Oral hygiene is a general term referring to any action of dental and gingival cleaning with the use of a toothbrush to disorganize oral biofilms [1]. Toothbrushing twice a day is generally considered as a key self-care behavior necessary to maintaining good oral health [2].

In sub-Saharan Africa (SSA), the first challenge in oral health is epidemiological [3]. The situation of children in these countries has degraded in the past decades, mainly due to the growing consumption of carbohydrate foods, poor tooth brushing habits, and limited access to dental services [4,5]. Lower education and occupation level of parents and rural residence were associated to higher caries values [6]. In 2019, more than 480 million people in the region were estimated to be suffering from oral conditions [7]. Overall prevalence of gingival bleeding is estimated at 75.7% and of dental caries among 5-year-olds at 46.3% [3,8,9].

SSA’s second challenge is for children who must manage their oral hygiene on a daily basis. While there is increasing awareness on oral hygiene, empirical evidence suggests that there is no concurrent increase in correct oral hygiene practice among key populations [10,11]. Children consistently assume poor oral hygiene compliance levels resulting in negative health consequences. Although twice-daily brushing associated with the regular application of fluoride toothpaste is widely recommended, low socio-economic and rural populations often brush less regularly [12]. In 2022, in spite of the efforts and commitments made in the last ten years, progress in the area of dental hygiene to reduce the morbidity associated with these diseases has been slow [13]. As for hand hygiene, these trends underscore the inadequacies of the educational approaches to oral hygiene promotion commonly adopted in SSA. Education must be the key favorable determinant for healthy oral hygiene practices, and improving oral health literacy interventions through basic health education should be promoted [14].

The WHO for Africa advanced digital solutions as the future of equitable, quality health care and resilient health systems [15]. Great strides have been made in boosting telemedicine, eLearning and mobile health. Mobile health technologies have the potential to make delivery of public health interventions more direct and efficient [16]. Mobile health apps are booming in Africa, which holds real hope for the continent in its quest for fairer and better-quality healthcare. The total number of mobile health programs implemented in SSA between 2006 and 2016 was 487. Of these, the eastern region with 17 countries and the western region with 16 countries had 287 and 145 mobile health programs, respectively. The eastern part of SSA shows high-high association for mobile health programs [17]. Recently, to contribute to better oral health for all, the WHO and the International Telecommunication Union have, in the context of the Be He@lthy Be Mobile initiative, developed a mOralHealth literacy module to improve the oral health literacy of individuals and communities [18].

Given the lack of research on mobile health apps in SSA, the objective of this study was to identify oral hygiene apps for children (OHAC) available on the Apple store and Android store and then have them qualitatively evaluated by OHP.

## 2. Materials and Methods

### 2.1. Design of the Study

This study analyzing OHAC accessible on mobile application stores in SSA was designed as a cross-sectional study. This study, which does not require regulatory approval, was conducted according to STROBE guidelines (Supplementary Table S1). The purpose of the study and its conduct were presented to all participants.

### 2.2. Selection of Oral Health Professionals

The following criteria were applied to select ten OHP (Supplementary Table S2). The inclusion criteria were: (i) to be an OHP, and/or (ii) to be or have been in a dental practice in SSA. The exclusion criteria were: (i) not owning a cell phone, (ii) unable to download applications from the iOS (App store) or Android (Google Play store) stores, (iii) never having used a mobile application, or, (iv) having hearing, visual, or motor disabilities.

### 2.3. Selection of the Oral Hygiene-Related Mobile Apps in the Senegal App Stores

The selection of OHAC was conducted by two university researchers (A.K. and A.D.) between 7–13 June 2022 in the African Apple Store and the African Google Play Stores. The words used for the search were “hygiène orale” (oral hygiene), “hygiène bucco-dentaire” (oral and dental hygiene), “santé orale” (oral health), and “brossage des dents” (tooth brushing). All the keywords were entered independently because there is no possibility of truncation or logical operators (AND, OR and NOT) in the iOS and Android stores.

The two researchers analyzed their lists of applications and eliminated duplicate applications (iOS or Android) and those that were only present in one of the two stores. The two researchers’ lists were then compared to ensure completeness. The remaining apps were then downloaded and the inclusion criteria checked: (1) oral hygiene topic, (2) French or English language, (3) for children, (4) available in both stores and (5) free apps at least for 7 days. Oral hygiene applications that require the addition of extra equipment (electric toothbrush, etc.), games, applications specific to a population (autism, etc.), shop of dental material, nutrition-related applications and examination training applications were excluded.

### 2.4. Evaluation of Children’s Mobile Applications Linked to Oral Hygiene in African App Stores

#### 2.4.1. Standardized Rating Scale for Mobile Applications

For the evaluation, the French version of the Mobile App Rating Scale (MARS-F) [19] was used [20]. The “app classification,” the initial step of the MARS scale, was examined by two university researchers. This scale is designed to rate mobile applications in the health care field. It consists of a main part that has 23 items organized into 5 sections (A, B, C, D, and E) and one additional section that has 6 items (section F) [20,21,22,23].

The engagement section (section A) has 5 items and analyzes if the application is interesting, fun, customizable and interactive (sends messages, alerts, feedback, reminders, allows sharing). The “features” section (section B) has 4 questions that focus on the application’s functionality, usability, learnability, ability to send reminders, messages, alerts, share information and feedback. The “aesthetics” section (section C) has 3 items and evaluates the overall visual appeal of the application, its graphic design, its color scheme and its stylish consistency. The “information quality” section (section D) has 7 items and assesses if the application includes information of high quality (text, references, comments and measurements) from a reliable source. The subjective section (section E) has 4 items and evaluates the interest of the user in the application. The final section of MARS (section F) asks the OHPs’ views on the potential impact of the screened applications on knowledge, potential shifts in the users’ attitudes and their intentions to engage in change, and the probability of changing “daily oral hygiene habits”.

A 5-point Likert scale (1 = strongly disagree and 5 = strongly agree) was used to evaluate each item. The mean score of the sections scores A, B, C, and D corresponds to the overall MARS quality score as reported in Stoyanov et al. [20]. The subjective quality score is the mean score of section E. The specificities of the app are the mean score of items of section F. A poor quality corresponds to the minimum score (1) and a high-quality score corresponds to a maximum score (5).

#### 2.4.2. Evaluation of the Children’s Mobile Applications Linked to Oral Hygiene

OHAC were rated by ten OHPs (Supplementary Table S2). Firstly, the raters were educated in the use of MARS-F scale. They watched a training video in French (obtainable on demand from the corresponding author) realized for the MARS-F scale [19] and based on the English training video by Stoyanov et al. [20]. This video presents with examples of each item and the answers. It also provides an exercise to evaluate a physical activity app. For this exercise, evaluators downloaded the app and then used it for at least 10 min before completing the MARS-F questionnaire. The raters then compared their scores with the video. If the individual rating score differed by 2 or more points, raters debated among themselves until they reached a consensus to guarantee a consistent understanding of the item.

Finally, raters evaluated the OHAC during the month of July 2022. For this, they downloaded all the included applications, tested them during 10 min and evaluated them thanks to a standardized online MARS-F questionnaire.

### 2.5. Statistical Analysis

Inter-rater reliability was determined by calculating intraclass correlations (ICC) (two-way random, mean measures, absolute agreement) [24,25]. The 95% confidence intervals were computed for every item, for every section and for the overall quality MARS-F score (sections A–D). Using the 95% confidence interval of the ICC estimate, values below 0.5 correspond to poor reliability, between 0.5 and 0.75 corresponds to moderate reliability, between 0.75 and 0.9 to good reliability, and those above 0.90 indicate excellent reliability [25]. Mean values and standard deviations were calculated for each item, for each section and for overall MARS-F quality score. Missing values (N/A: not applicable) caused item 19 to be excluded from all analyses, and the mean of section D was adjusted accordingly.

Box plots were produced to compare the differences between the quality of the applications, per item and per section.

To provide an overview of the mean scores by item (row) and by application (column), a heat map was produced. The gradations of color showed if the score was close to 1 (low, red), or close to 5 (high, green).

Pearson’s coefficient (r) was calculated to analyze the correlation between the average MARS-F quality and subjective item 23 (“What is your overall star rating of the app?”). In order to indicate the popularity of the apps, the number of stars given by users as well as the number of reviewers in the iOS and Android stores was reported.

Statistical analyses were carried out with R using the “dplyr”, “psych”, and “ggplot2” packages from the R Project for Statistical Computing (Version 4.1.1. 10 August 2021).

## 3. Results

### 3.1. Characteristics of Oral Health Professionals

The characteristics of the OHPs are described in Supplementary File Table S2. Their years of experience as oral health professionals ranged from 2 to 41 years with a median of 15 years. Five OHPs had hospital activity, three OHPs had hospital and liberal activity and two OHPs had hospital and university activity. Two OHPs were generalists, five OHPs were public health specialists, two OHPs were prosthodontists and one OHP was a periodontist.

### 3.2. Selection of the Oral Hygiene-Related Mobile Apps for Children

**Figure 1:**
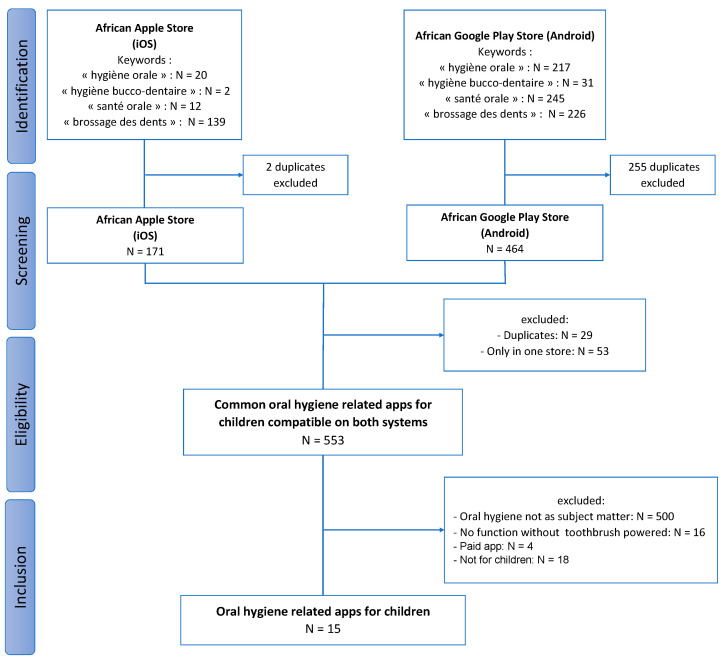
Flowchart of the section of oral hygiene-related mobile applications for children available in Sub-Saharan Africa.

Searching with the keywords yielded 171 apps in the App Store and 464 apps in the Google Play Store (Figure 1). In each list, duplicates were removed. The lists were pooled and an analysis of the app name and developer was performed. A list of 553 apps available in the 2 stores was established. After analyzing theses apps, 500 were eliminated because their main topic was not oral hygiene, 18 because they were not suitable for children, 16 because they needed an electric toothbrush to work and 4 because they were paid. Finally, 15 apps were included.

### 3.3. Characteristics of the Oral Hygiene-Related Mobile Apps for Children Available in Sub-Saharan Africa

The Supplementary Tables S3–S9 provide technical and descriptive information about OHAC. The 15 included applications all had different developers. Ten apps were completely free and 5 needed in-app purchases.

The description of the 15 OHAC are presented in Table 1. OHAC were oriented toward behavior change (15/15, 100%), goal-setting (15/15, 100%), increasing happiness or wellbeing (12/15, 80.0%), on entertainment (12/15, 80.0%), and on relationship (10/15, 66.7%). In order for the users to reach the determined objectives the applications used mainly, theoretical background or strategies, information and education (15/15, 100%), CBT–behavioral (15/15, 100%) and goal setting (14/15, 93.3%). All applications were usable by children and 5 were specific for children under 12 years old. Only one application allowed sharing, one allowed password protection, one send reminders, three sent reminders and two needed web access to function.

Three of the fifteen applications had mini cartoons that feature a character who brushes his teeth (*Ben le Koala*, *Mimizaur se brosse les dents* and Chomper Chums). Three apps needed an active participation of the child by helping the main character to brush his teeth (*Bonne nuit Caillou*, *Brosse à dents* and *Mon Raccoon*). Two apps use the phone’s camera to transform the child into a knight or a Pokemon who will fight against the monsters that are bacteria thanks to his brushing (Brushing Hero and Pokemon Smile). Three applications were timers and provided guidance on the area of the teeth to be brushed (Brush jam, Disney Magic Timer by Oral-B and BrushYourTeeth) and four were timers only (Brush DJ, Brushing time, *De belles dents* and Happy kids Timer).

### 3.4. Reliability of the Evaluation

The reliability was good for information quality (section D) (ICC 0.74, 95% CI 0.65–0.81) and for the subjective quality (section E) (ICC 0.61, 95% CI 0.44–0.74). The reliability was poor for engagement (section A) (ICC 0.51, 95% CI 0.33–0.66), for functionality (section B) (ICC 0.14, 95% CI 0–0.43), and aesthetics (section C) (ICC 0.03, 95% CI 0–0.40), and for the section on specificities (section F) (ICC 0.24, 95% CI 0–0.45).

### 3.5. Qualitative Evaluation of Oral Hygiene-Related Mobile Apps for Children Available in Sub-Saharan Africa

#### 3.5.1. Overall Quality MARS-F Scores of Oral Hygiene-Related Mobile Apps for Children Available in Sub-Saharan Africa

**Figure 2:**
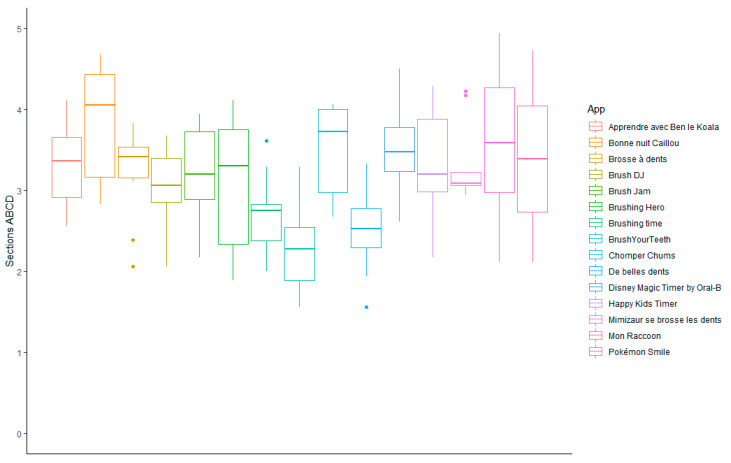
Overall MARS-F qualitative evaluation of oral hygiene-related mobile apps for children available in Sub-Saharan Africa. Section A: engagement; section B: functionality; section C: aesthetics; section D: information.

The highest overall quality MARS-F scores (sections A–D) (Figure 2 and Supplementary Tables S10 and S11) were achieved by *Bonne nuit Caillou* (3.89 ± 0.74), *Mon Raccoon* (3.63 ± 0.95), and Chomper Chums (3.54 ± 0.54); while the lowest scores were obtained by Brushing time (2.31 ± 0.61), *De belles dents* (2.55 ± 0.55) and Brushing Hero (2.77 ± 0.53).

**Figure 3:**
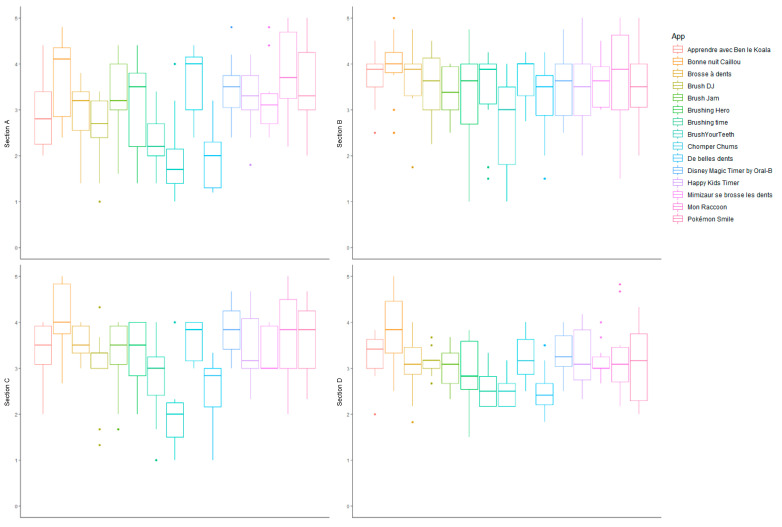
Qualitative evaluation of oral hygiene-related mobile apps for children available in Sub-Saharan Africa. Section A: engagement; section B: functionality; section C: aesthetics; section D: information.

The scores obtained for engagement (section A), functionality (section B), aesthetics (section C) and information quality (section D) are presented in Figure 3. The engagement scores varied from 1.94 ± 0.67 for *De belles dents* to 3.84 ± 0.95 for *Mon Raccoon*. The functionality scores varied from 2.70 ± 1.05 for Brushing time to 3.95 ± 0.74 for *Bonne nuit Caillou*. The aesthetics scores varied from 2.03 ± 0.82 for Brushing time to 4.07 ± 0.81 for *Bonne nuit Caillou*. The information quality scores varied from 2.50 ± 0.35 for Brushing time to 3.82 ± 0.80 for *Bonne nuit Caillou*.

#### 3.5.2. Subjective Qualitative Evaluation of Oral Hygiene-Related Mobile Apps for Children Available in Sub-Saharan Africa

**Figure 4:**
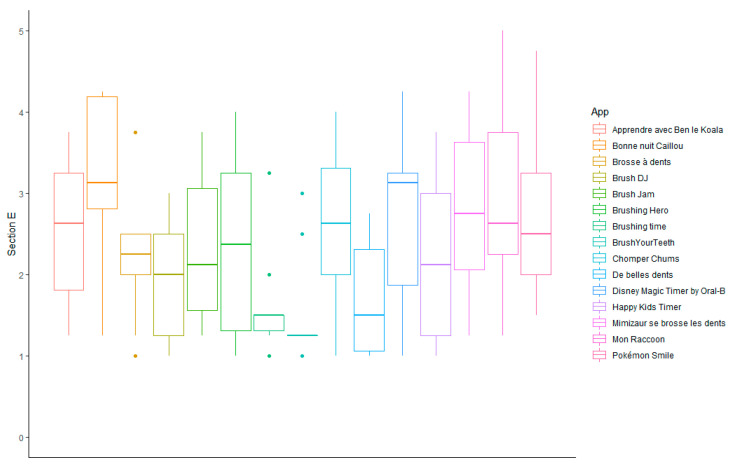
Subjective qualitative evaluation of oral hygiene-related mobile apps for children available in Sub-Saharan Africa (section E).

OHAC available in Sub-Saharan Africa had subjective quality scores (section E) ranging from 1.50 ± 0.68 for Brushing time to a mean of 3.25 ± 0.97 for *Bonne nuit Caillou* (Figure 4).

### 3.6. Evaluation of the Specific Content Oral Hygiene-Related Mobile Apps for Children Available in Sub-Saharan Africa

**Figure 5:**
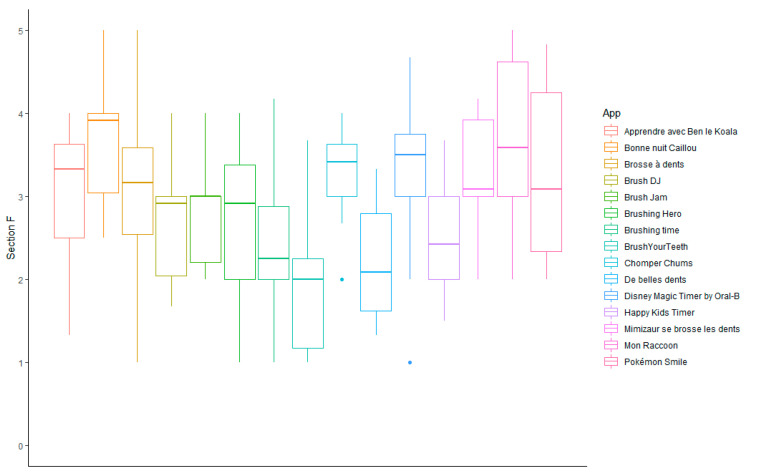
Specificity scores of oral hygiene-related mobile apps for children available in Sub-Saharan Africa (section F).

The Figure 5 presents the specificity scores (section F) of each app (Figure 5). The scores ranged from 1.95 ± 0.88 for Brushing time to 3.75 ± 0.84 for *Bonne nuit Caillou*. *Bonne nuit Caillou*, *Mon Raccoon* (3.60 ± 1.14) and Pokémon smile (3.32 ± 1.07) obtained the best specificity scores. Brushing time (1.95 ± 0.88), *De belles dents* (2.23 ± 0.69) and Brushing hero (2.42 ± 0.95) obtained the worst specificity scores and were the only apps to have mean specificity scores lower than 2.5.

For the 15 apps included, the overall quality MARS-F score (sections A–D) was found to be consistently superior to the specificity score (section F), that was consistently inferior to the subjective quality score (section E) (Tables S10 and S11).

### 3.7. Strengths and Limitation of Oral Hygiene-Related Mobile Apps for Children Available in Sub-Saharan Africa

The strengths and the weakness of each OHAC included in this study are analyzed in the heatmap (Table 2). The functionality score was the strength of the quality score for ten apps, the aesthetic score was the strength for four apps (*Bonne nuit Caillou,* BrushYourTeeth, Disney magic Timer by Oral-B and Pokémon smile) and the engagement was the strength for *Mon Racoon*.

Information (section D) was the low point in the quality score for eight applications (Brush jam, BrushYourTeeth, Chomper Chums, Disney magic Timer by Oral-B, Happy kids Timer—Matin, *Mimizaur se brosse les dents*, *Mon Raccoon* and Pokémon smile), whereas the engagement (section A) was the weakness for seven apps (*Apprendre avec Ben le Koala, Brosse à dents*, Brush DJ, Brushing Hero, Brushing time and, *De belles dents*). For all applications except *Brosse à dents* and *De belles dents*, the lowest scores in the MARS-F overall quality score (sections A–D) were obtained for the item 18 (credibility). *Brosse à dents* obtained the lowest score for customization (item 3). *De belles dents* obtained the lowest score for interactivity (item 4).

Regarding the subjective quality score (section E), the overall star rating (item 23) scored the highest for all applications and item 22, indicating if people would be interested in paying for this application, scored the lowest.

For the specificity scores (section F), all except five applications (Brushing Hero, Brushing time, BrushYourTeeth, *De belles dents*, Happy Kids Timer), scored above 2.5 for all items.

### 3.8. Comparison of the MARS-F Score of Oral Health Professionals and the Star Rating of Raters in Stores

The correlation between the mean overall quality MARS-F score (sections A–D) and the MARS-F overall star rating (item 23: “What is your overall star rating of the app?”) was good (*r* = 0.79, *p <* 0.001). MARS-F overall star rating was inferior to the overall quality MARS-F score for the 15 OHAC studied. Three apps (Brush DJ, Brush jam and Pokémon Smile) were reviewed in the iOS store and by only one person. Two apps (*Brosse à dents* and Happy kids Timer—Matin) were reviewed in the Android store and obtained higher scores than the MARS-F overall star rating (item 23) and the overall quality MARS-F score (Table 3).

## 4. Discussion

In children, efforts to improve tooth brushing have relied on health education, based on the premise that inappropriate knowledge is the main constraint. However, Werner et al. concluded, in a systematic review, that health education alone does not significantly enhance toothbrushing behaviors or attitudes [26]. Most OHPs know that tooth brushing is important, but lack the self-efficacy and other skills to enforce tooth brushing habits [27]. In Rwanda, for example, children who did not use a toothbrush (62.7%) or toothpaste (70.0%) and cleaned their teeth less than once a day (55.3%) had a higher prevalence of untreated caries. About one-third of people living in rural areas cleaned their teeth once a day or more, compared to two-thirds of people living in urban areas (35.4% vs. 71.2%) [28]. In SSA, the contextual, geographical, economic, cultural or behavioral influences should be considered. So, different oral hygiene practices are used to overcome endemic diseases such as dental caries and oral infections. In Mali for instance, natural plant-based toothbrushes are used for eliminating bacterial biofilms [29]. Traditional chewing sticks have served as the primary form of dental care for rural communities in resource-poor settings for millennia [30]. Similarly, the health situation with unequal distribution of oral health professionals (OHPs) and the lack of appropriate facilities affects access to dental care with 90% of dental problems not being treated [31].

Thus, OHAC could help to improve toothbrushing habits [32] and especially in Africa where caries remains a public health problem [3]. Electronic mobile technologies such as smartphones and applications (apps) have become an integral part of society [33]. Smartphone ownership is fast growing in SSA, where in 2015 it was highest in South Africa, and ranged between 30–35% for Kenya, Nigeria, Senegal and Ghana [34]. Young people were the most frequent users. SSA has 456 million mobile phone users, a phone penetration rate of just 44% compared with 66% worldwide in 2018 [35]. With a young population at ease with mobile accounts, apps for shopping, and healthcare, that number is expected to rise [35]. However, while significant advances have been achieved in mobile connectivity, widespread Internet access remains elusive. In developing countries, less than 45% of the population is connected, while in the least developed countries, only 20% is actually connected. Many people in Sub-Saharan Africa are potentially at risk of exclusion, in particular people living in rural areas, women, adolescents, children and deprived communities [36,37].

To our knowledge, no study has investigated OHAC disponible in Sub-Saharan Africa. Screening of these apps in the African iOS and Android stores resulted in the inclusion of fifteen apps. A previous study, conducted in France and focusing on OHAC, analyzed nine apps [38]. Among these apps, two were designed for children. These two apps (Disney Magic Timer by Oral-B and *Mimizaur se brosse les dents*) were also analyzed in our study. Four other studies also looked at applications related to oral hygiene but did not focus on children. The first was conducted in the United States and included 33 apps in English [39]. The next two were conducted in United Kingdom and included 20 patient-focused oral hygiene applications [40,41]. The last was conducted in Australia and included 18 applications centered on self-management behaviors for dental caries prevention, such as dietary intake, oral hygiene, and fluoride use [42].

While all included applications addressed oral hygiene in children, strategies diverged. Twelve of the 15 applications studied were based on the principle of controlling brushing time of the teeth which varied from 2 to 3 min depending on the applications. Five of these applications were timers with music (Brush DJ, Brush jam and BrushYourTeeth) or not (Brushing time and *De belles dents*). Five others stage characters (Disney magic Timer by Oral-B, *Mimizaur se brosse les dents* and Chomper Chums) or make the child a character (Pokémon Smile or Brushing Hero). Happy kids Timer—Matin was a timer that helps the child to perform all the actions of the morning including brushing teeth. Two applications (My Raccoon and *Apprendre avec Ben le Koala*) via a main character teach the child several gestures of daily life including the ritual of brushing teeth (toothbrush, toothpaste, brushing, duration…). The last two applications included (*Bonne nuit Caillou* and *Brosse à dents*) were not timers but required active participation of the child. *Bonne nuit Caillou* was not limited to the ritual of brushing teeth but included various actions such as eating. Although these apps used different strategies, they all had overall MARS-F quality scores above 2.5 except Brushing time which scored 2.31. Some of the apps indicated to the child the area to be cleaned without explanation of the technique. This part of the toothbrushing should be taken into account. A recent study demonstrated that adolescents aged 10 to 15 unequally divided their brushing time between external, internal and occlusal surfaces. Notably, cleaning of internal surfaces represented a maximum of 15.8% of overall brushing time [43]. The application Chomper Chums presented and allowed the timing of other oral hygiene techniques such as flossing and using mouthwash but without explanation. Only the app Mon Raccoon addressed the choice of food. This result was consistent with that of Tiffany et al. (2018) who studied U.S. mobile applications for oral health promotion and concluded that when diet was addressed in the application, content was usually short and could run counter to the goal of improving user health behavior [44].

Depending on the country, the results of the application evaluations differed, although the same MARS-F evaluation scale was used. For example, Disney Magic Timer by Oral-B performed similarly in France and Sub-Saharan Africa (3.70 ± 0.30 vs. 3.52 ± 0.59 as overall MARS-F score). Conversely, different results were observed for *Mimizaur se brosse les dents.* French OHPs rated it 2.8 ± 0.22, whereas African OHPs 3.34 ± 0.47.

Studies have shown that healthcare professionals and users evaluate apps differently because they have different perspectives [38,45,46]. First, for users, star ratings as well as user comments are valuable because they allow for an assessment of the effectiveness and popularity of applications [47]. In the African app stores, few evaluations of OHAC are available. Only three applications (Brush DJ, Brush jam and Pokémon smile) have been reviewed and only by one reviewer in the African Apple store and two applications (*Brosse à dents* and Happy kids Timer—Matin) have been reviewed by more than 20,000 people in the African Google Play store. Applications included in this study were evaluated in stores in other countries. Disney Magic Timer by Oral-B had been rated by 974 users in the French Apple store and by 55,000 users in the French Android store [38]. *Mimizaur se brosse les dents* obtained 4.5 stars by 22 raters in the Apple store and 4.6 stars by 661 raters in the Android store [38]. The star ratings by users do not allow an objective assessment of quality. For example, *Brosse à dents* and Happy kids Timer—Matin were rated 4.3 and 4.4 stars in the Google Play store, respectively, whereas they obtained overall MARS-F score of 3.29 ± 0.55 and 3.35 ± 0.69. Conversely, Brush DJ, Brush jam and Pokémon smile had lower star ratings than the overall MARS-F score. Thus, the number of raters is an important factor to consider for the relevance of star ratings in stores. Second, users and professionals rate apps differently even when using different validated scales. In the study of Fijačko et al. (2020), several apps were evaluated using uMARS [48]. Brush DJ, Brushing hero, Chomper Chums, Disney Magic Timer by Oral-B, Mimizavr Clean Teeth (equivalent to *Mimizaur se brosse les dents* in the African stores), obtained overall MARS quality scores of 3.9, 4.6, 4.4, 4.3 and 4.8, respectively. In our study, they obtained lower overall quality MARS-F score by African OHPs (3.06, 2.77, 3.54, 3.52 and 3.34). This difference in scoring between OHPs and users may have different reasons. Although similar, the MARS and uMARS questionnaires [49] have minor discrepancies. For instance, the information section of MARS consists of seven questions while uMARS has only four. The credibility item is based on publications in the case of MARS and on users’ perceptions for uMARS. Furthermore, professionals focus on the content of the application while users focus on attractiveness, design, or gamification.

OHPs felt that the majority of apps tested except Brushing Hero, Brushing time and *De belles dents* had the potential to positively influence user knowledge, attitudes, and intentions to change, as well as the likelihood of actual change in oral hygiene behavior. At the same time, they stated that they would recommend these applications to children who needed them. These results are different from those obtained in France [38]. Indeed, although OHPs thought that oral hygiene applications could change behavior, they stated that they would not recommend them. Another study, conducted in France, had obtained similar conclusions with applications related to nutrition [46]. Thus, social-cultural differences must be taken into consideration.

Concerning the strength of oral-hygiene-related mobile apps, two previous studies [38,40] compared oral hygiene apps using the MARS scale and concluded that the strongest point was their functionality as five of the fifteen apps tested in our study. The weakness of eight of the fifteen apps tested was the information as in the study of Sharif et al. [40] and for five of the nine apps in the study of Carrouel et al. [38]. The credibility of the application was the main issue. OHPs identified the source of the information, but in fact remained suspicious of its accuracy or credibility. Furthermore, reviewers encountered problems scoring the degree of scientific proof and thus selected “N/A The application has not been tested” for the majority of applications. To our knowledge, only five of the fifteen apps (Brush DJ [48,50,51,52], Brushing hero [48], Chomper Chums [48], Disney Magic Timer by Oral-B [38,42,48], and *Mimizaur se brosse les dents* [38,48]) are referenced in PubMed. To enhance the credibility of apps and consequently the level of information, new scientific studies are needed. Clinical effectiveness must be proven through randomized controlled trials. At the same time, economic studies must be carried out to evaluate a possible benefit [53]. Although OHPs seemed to be in favor of the use of OHAC, the ethical aspects regarding the use of personal health data and the implementation of applications for commercial reasons must not be forgotten. Furthermore, it is important to note that the use of these mobile applications represents a real hope for improving oral health provided that the entire population has access to these applications, including the most disadvantaged.

Several limitations exist in this study. First, in this study, the evaluation was carried out using the MARS scale, which is the most widely reported in the published literature. But other scales for evaluating health applications such as ENLIGHT [54] could have been used. Second, the search for applications was conducted in the Apple and Android stores, whereas other stores, such as the Huawei store, the Windows Phone Store, and the Samsung store exist. Third, OHPs carried out the evaluation of these applications intended for the general public. It would therefore be interesting to have these apps evaluated by users via the user-specific MARS scale [49] and to compare the results. Fourth, inter-rater reliability for several sections was low. Fifth, MARS-F only allowed to know if the application was adapted to children under 12 years old but it would have been interesting to know more precisely if some applications were intended for very young children.

## 5. Conclusions

This study indicated that OHPs positively evaluated the quality of the majority of OHAC, their impact on user knowledge, attitudes, and intentions to change, and the probability of actual oral hygiene behavior modification. They stated that they would recommend their use to their patients who need them. However, studies analyzing the change in oral hygiene behavior of children using these apps need to be conducted.

## Figures and Tables

**Table 1:** Description of the twenty oral hygiene-related mobile applications for children.

Characteristic	App (*n* = 15), *n* (%) ^1^
**Focus or target**	
Increase happiness or wellbeing	12 (80.0)
Behavior change	15 (100)
Goal-setting	15 (100)
Entertainment	12 (80.0)
Relationship	10 (66.7)
**Theoretical background or strategies**	
Information/Education	15 (100)
Monitoring/Tracking	6 (40.0)
Goal-setting	14 (93.3)
Advice/Tips/Strategies/Skills training	10 (66.7)
CBT–Behavioral (positive events)	15 (100)
Gratitude	8 (53.3)
**Age group**	
Children (under 12 years)	15 (100)
Adolescents (13–17 years)	5 (33.3)
Young adults (18–25 years)	5 (33.3)
Adults	5 (33.3)
**Technical aspects of app**	
Allows sharing (Facebook, Twitter, etc.)	1 (6.7)
Allows password-protection	1 (6.7)
Sends reminders	1 (6.7)
Needs web access to function	2 (13.3)

^1^ More than one could be applicable; therefore, percentages do not add to 100%.

**Table 2:** Heatmap of the mean scores per application and per item. The colors are linked to the scores and range from red (1: worst score) to green (5: best score).

	*Apprendre avec Ben le Koala*	*Bonne nuit Caillou*	*Brosse à dents*	Brush DJ	Brush Jam	Brushing Hero	Brushing time	BrushYourTeeth	Chomper Chums	*De belles dents*	Disney Magic Timer by Oral-B	Happy Kids Timer	*Mimizaur se brosse les dents*	*Mon Raccoon*	Pokémon Smile
Section A															
Item 1—Entertainment	3.4	4.2	2.9	2.3	3.6	3.7	2.3	2.3	4.0	1.9	3.9	3.3	3.6	3.8	3.8
Item 2—Interest	3.4	4.0	3.3	2.7	3.5	3.6	2.2	2,10	4.0	2.3	3.8	3.3	3.7	4.0	3.8
Item 3—Customization	2.4	2.9	2.0	2.7	3.2	2.2	2.4	1.5	3.3	1.8	3.3	2.9	2.6	3.8	3.3
Item 4—Interactivity	2.3	3.4	2.7	2.5	2.7	2.7	1.8	1.6	3.2	1.5	2.7	3.3	2.5	3.5	3.3
Item 5—Target group	3.3	4.2	3.7	2.6	3.6	3.0	3.0	2.5	3.5	2.2	3.6	3.4	3.8	4.1	3.4
Section B															
Item 6—Performance	3.4	3.8	3.8	3.9	3.4	3.4	3.4	2.9	3.6	3.4	3.7	3.8	3.7	3.6	3.5
Item 7—Ease of use	3.6	3.7	3.6	3.4	3.2	3.4	3.5	2.6	3.8	3.3	3.2	3.4	3.2	3.5	3.5
Item 8—Navigation	3.9	4.1	3.7	3.6	3.5	3.2	3.3	2.8	3.9	3.3	3.4	3.6	3.7	3.9	3.5
Item 9—Gestural design	3.9	4.2	3.5	3.1	3.3	3.1	3.4	2.5	3.5	3.0	3.7	3.2	3.8	3.8	3.5
Section C															
Item 10—Layout	3.7	4.0	4.0	3.5	3.5	3.1	3.4	1.9	3.7	3.1	3.6	3.6	3.4	3.8	3.6
Item 11–Graphics	3.2	4.1	3.3	2.9	3.2	3.5	2.6	2.0	3.7	2.3	4.0	3.4	3.3	3.6	3.7
Item 12—Visual appeal	3.3	4.1	3.3	2.7	3.1	3.3	2.3	2.2	3.5	2.1	3.9	3.3	3.4	3.7	3.6
Section D															
Item 13—Accuracy	3.8	4.1	3.3	3.5	3.1	3.0	2.8	2.2	3.8	2.4	3.7	3.5	3.6	3.6	3.5
Item 14—Goals	3.3	4.0	3.2	2.9	3.1	3.1	3.0	3.0	3.5	3.0	3.3	3.0	3.0	3.2	2.8
Item 15—Quality of information	3.2	3.8	3.3	3.2	3.2	2.9	2.7	2.6	3.2	2.5	3.3	3.5	3.4	3.5	3.6
Item 16—Quantity of information	3.5	4.1	2.9	3.3	3.1	2.9	2.8	2.6	3.3	2.4	3.5	3.5	3.3	3.3	3.2
Item 17—Visual information	3.8	4.4	3.3	3.9	3.8	3.7	2.8	3.1	3.4	2.8	3.6	3.7	3.6	3.5	3.6
Item 18—Credibility	1.9	2.5	2.3	2.0	1.9	1.8	1.4	1.5	2.2	2.0	2.3	2.1	1.9	2.5	1.9
Section E															
Item 20—Recommendations	2.8	3.6	2.6	2.3	2.6	2.4	1.5	1.5	2.8	1.6	2.8	2.3	3.0	3.0	2.5
Item 21—Usage	2.6	3.3	2.4	2.0	2.3	2.7	1.6	1.5	2.7	1.7	3.0	2.2	2.8	2.9	2.6
Item 22—Price	1.8	2.4	1.4	1.2	1.6	1.6	1.0	1.2	1.6	1.4	1.8	1.8	2.0	2.0	2.0
Item 23—Overall rating	3.1	3.7	2.9	2.4	2.7	2.7	2.3	1.8	3.2	2.0	3.4	2.7	3.1	3.6	3.6
Section F															
Awareness	3.1	3.8	3.3	2.6	2.7	2.6	2.4	2.0	3.4	2.2	3.3	2.7	3.3	3.6	3.3
Knowledge	3.1	3.7	3.1	2.8	2.9	2.4	2.5	1.8	3.4	2.5	3.1	2.2	3.2	3.6	3.3
Attitude	3.0	3.8	2.8	2.8	2.9	2.6	2.4	1.8	3.2	2.3	3.4	2.7	3.2	3.6	3.4
Intention to change	2.9	3.8	3.0	2.7	2.8	2.8	2.3	2.0	3.2	2.3	3.3	2.8	3.4	3.6	3.4
Help seeking	3.2	3.6	2.7	2.6	2.7	2.5	2.4	2.1	3.0	1.7	3.1	2.3	3.2	3.6	3.1
Behaviour change	3.1	3.8	3.2	3.0	2.7	2.6	2.5	2.0	3.4	2.4	3.3	2.4	3.3	3.6	3.4

**Table 3:** MARS overall star rating, overall quality MARS score, star rating in the iOS App Store, and star rating in the Android app store of the fifteen oral hygiene-related mobile apps for children. NA: Not applicable.

App Name	MARS-F Overall Star Rating (Item 23)	Overall Quality MARS-F Score	Star Rating in the iOS App Store (No. of Raters)	Star Rating in the Android App Store (No. of Raters)
*Apprendre avec Ben le Koala*	3.10 ± 0.88	3.33 ± 0.51	NA	NA
*Bonne nuit Caillou*	3.70 ± 0.82	3.89 ± 0.74	NA	NA
*Brosse à dents*	2.90 ± 1.10	3.29 ± 0.55	NA	4.3 (24,855)
Brush DJ	2.40 ± 0.70	3.06 ± 0.52	3 (1)	NA
Brush jam	2.70 ± 0.95	3.24 ± 0.59	3 (1)	NA
BrushYourTeeth	2.70 ± 1.16	3.13 ± 0.86	NA	NA
Brushing Hero	2.30 ± 1.16	2.77 ± 0.53	NA	NA
Brushing time	1.80 ± 0.79	2.31 ± 0.61	NA	NA
Chomper Chums	3.20 ± 1.03	3.54 ± 0.54	NA	NA
*De belles dents*	2.00 ± 0.94	2.55 ± 0.55	NA	NA
Disney magic Timer by Oral-B	3.40 ± 1.17	3.52 ± 0.59	NA	NA
Happy kids Timer—Matin	2.70 ± 1.06	3.35 ± 0.69	NA	4.6 (20,133)
*Mimizaur se brosse les dents*	3.10 ± 0.99	3.34 ± 0.47	NA	NA
*Mon Raccoon*	3.60 ± 1.26	3.63 ± 0.95	NA	NA
Pokémon Smile	3.60 ± 0.84	3.44 ± 0.82	1 (1)	NA

## Data Availability

The data presented in this study are available on request from the corresponding author.

## Supplementary Materials

The following supporting information can be downloaded at: https://www.mdpi.com/article/10.3390/ijerph191912565/s1, Table S1: STROBE Statement—checklist of items that should be included in reports of observational studies; Table S2: Characteristics of the raters, the hardware, and the software used; Table S3: Developer, Scare rating and Paid content of the 15 oral-health-related mobile applications, for children, included in the study; Table S4: Brief description of the 15 oral-health-related mobile applications, for children, included in the study; Table S5: Targets of the 15 oral-health-related mobile applications, for children, included in the study; Table S6: Theoretical background and strategies of the 15 oral-health-related mobile applications, for children, included in the study; Table S7: Affiliations of the 15 oral-health-related mobile applications, for children, included in the study; Table S8: Age group of the 15 oral-health-related mobile applications, for children, included in the study; Table S9: Technical aspects of the 15 oral-health-related mobile applications, for children, included in the study; Table S10: Mobile App Rating Scale-French (MARS-F) scoring by section for the 15 oral-health-related mobile applications, for children, included in the study; Table S11: Mobile App Rating Scale-French (MARS-F) scoring by items the 15 oral-health-related mobile applications, for children, included in the study.

## References

[1] Bourgeois D., Saliasi I., Dussart C., Llodra J.C., Tardivo D., Laforest L., Bravo M., Viennot S., Foti B., Carrouel F. (2018). Educational Outcomes of a New Curriculum on Interproximal Oral Prophylaxis for Dental Students. PLoS ONE.

[2] Raison M.H., Corcoran R., Burnside G., Harris R. (2020). Oral Hygiene Behaviour Automaticity: Are Toothbrushing and Interdental Cleaning Habitual Behaviours?. J. Dent..

[3] Teshome A., Muche A., Girma B. (2021). Prevalence of Dental Caries and Associated Factors in East Africa, 2000–2020: Systematic Review and Meta-Analysis. Front. Public Health.

[4] Tinanoff N., Baez R.J., Diaz Guillory C., Donly K.J., Feldens C.A., McGrath C., Phantumvanit P., Pitts N.B., Seow W.K., Sharkov N. (2019). Early Childhood Caries Epidemiology, Aetiology, Risk Assessment, Societal Burden, Management, Education, and Policy: Global Perspective. Int. J. Paediatr. Dent..

[5] Elamin A., Garemo M., Mulder A. (2021). Determinants of Dental Caries in Children in the Middle East and North Africa Region: A Systematic Review Based on Literature Published from 2000 to 2019. BMC Oral Health.

[6] Peters A., Brandt K., Wienke A., Schaller H.-G. (2022). Regional Disparities in Caries Experience and Associating Factors of Ghanaian Children Aged 3 to 13 Years in Urban Accra and Rural Kpando. Int. J. Environ. Res. Public Health.

[7] GBD Results. https://vizhub.healthdata.org/gbd-results.

[8] Global Burden of Disease (2015). Study 2013 Collaborators Global, Regional, and National Incidence, Prevalence, and Years Lived with Disability for 301 Acute and Chronic Diseases and Injuries in 188 Countries, 1990–2013: A Systematic Analysis for the Global Burden of Disease Study 2013. Lancet.

[9] Osuh M.E., Oke G.A., Lilford R.J., Owoaje E., Harris B., Taiwo O.J., Yeboah G., Abiona T., Watson S.I., Hemming K. (2022). Prevalence and Determinants of Oral Health Conditions and Treatment Needs among Slum and Non-Slum Urban Residents: Evidence from Nigeria. PLOS Glob. Public Health.

[10] Duangthip D., Chu C.H. (2020). Challenges in Oral Hygiene and Oral Health Policy. Front. Oral Health.

[11] Fantaye W., Nur A., Kifle G., Engida F. (2022). Oral Health Knowledge and Oral Hygiene Practice among Visually Impaired Subjects in Addis Ababa, Ethiopia. BMC Oral Health.

[12] Waldron C., Nunn J., Mac Giolla Phadraig C., Comiskey C., Guerin S., van Harten M.T., Donnelly-Swift E., Clarke M.J. (2019). Oral Hygiene Interventions for People with Intellectual Disabilities. Cochrane Database Syst. Rev..

[13] Oral Health. https://www.afro.who.int/health-topics/oral-health.

[14] Diendéré J., Ouattara S., Kaboré J., Traoré I., Zeba A.N., Kouanda S. (2022). Oral Hygiene Practices and Their Sociodemographic Correlates among Adults in Burkina Faso: Results from the First National Survey. BMC Oral Health.

[15] Health Technologies and Innovations. https://www.afro.who.int/programmes-clusters/HTI.

[16] Meyer A.J., Armstrong-Hough M., Babirye D., Mark D., Turimumahoro P., Ayakaka I., Haberer J.E., Katamba A., Davis J.L. (2020). Implementing MHealth Interventions in a Resource-Constrained Setting: Case Study From Uganda. JMIR mHealth uHealth.

[17] Lee S., Cho Y., Kim S.-Y. (2017). Mapping MHealth (Mobile Health) and Mobile Penetrations in Sub-Saharan Africa for Strategic Regional Collaboration in MHealth Scale-up: An Application of Exploratory Spatial Data Analysis. Glob. Health.

[18] World Health Organization (2021). Mobile Technologies for Oral Health: An Implementation Guide.

[19] Saliasi I., Martinon P., Darlington E., Smentek C., Tardivo D., Bourgeois D., Dussart C., Carrouel F., Fraticelli L. (2021). Promoting Health via MHealth Applications Using a French Version of the Mobile App Rating Scale: Adaptation and Validation Study. JMIR mHealth uHealth.

[20] Stoyanov S.R., Hides L., Kavanagh D.J., Zelenko O., Tjondronegoro D., Mani M. (2015). Mobile App Rating Scale: A New Tool for Assessing the Quality of Health Mobile Apps. JMIR mHealth uHealth.

[21] Grainger R., Townsley H., White B., Langlotz T., Taylor W.J. (2017). Apps for People with Rheumatoid Arthritis to Monitor Their Disease Activity: A Review of Apps for Best Practice and Quality. JMIR mHealth uHealth.

[22] Masterson Creber R.M., Maurer M.S., Reading M., Hiraldo G., Hickey K.T., Iribarren S. (2016). Review and Analysis of Existing Mobile Phone Apps to Support Heart Failure Symptom Monitoring and Self-Care Management Using the Mobile Application Rating Scale (MARS). JMIR mHealth uHealth.

[23] Salazar A., de Sola H., Failde I., Moral-Munoz J.A. (2018). Measuring the Quality of Mobile Apps for the Management of Pain: Systematic Search and Evaluation Using the Mobile App Rating Scale. JMIR mHealth uHealth.

[24] Shrout P.E., Fleiss J.L. (1979). Intraclass Correlations: Uses in Assessing Rater Reliability. Psychol. Bull..

[25] Koo T.K., Li M.Y. (2016). A Guideline of Selecting and Reporting Intraclass Correlation Coefficients for Reliability Research. J. Chiropr. Med..

[26] Werner H., Hakeberg M., Dahlström L., Eriksson M., Sjögren P., Strandell A., Svanberg T., Svensson L., Wide Boman U. (2016). Psychological Interventions for Poor Oral Health: A Systematic Review. J. Dent. Res..

[27] Huebner C.E., Riedy C.A. (2010). Behavioral Determinants of Brushing Young Children’s Teeth: Implications for Anticipatory Guidance. Pediatr. Dent..

[28] Hackley D.M., Jain S., Pagni S.E., Finkelman M., Ntaganira J., Morgan J.P. (2021). Oral Health Conditions and Correlates: A National Oral Health Survey of Rwanda. Glob. Health Action.

[29] Sogodogo E., Doumbo O., Kouriba B., Aboudharam G. (2021). Microbial Biodiversity of Natural Toothbrushes in Mali. New Microbes New Infect..

[30] Nyambe M.M., Kwembeya E.G., Lisao K., Hans R. (2021). Oral Hygiene in Namibia: A Case of Chewing Sticks. J. Ethnopharmacol..

[31] (2016). Regional Committee for Africa, 66 Regional Oral Health Strategy 2016–2025: Addressing Oral Diseases as Part. of NCDs (Document AFR/RC66/5).

[32] Jacobson D., Jacobson J., Leong T., Lourenco S., Mancl L., Chi D.L. (2019). Evaluating Child Toothbrushing Behavior Changes Associated with a Mobile Game App: A Single Arm Pre/Post Pilot Study. Pediatr. Dent..

[33] Reddy P., Dukhi N., Sewpaul R., Ellahebokus M.A.A., Kambaran N.S., Jobe W. (2021). Mobile Health Interventions Addressing Childhood and Adolescent Obesity in Sub-Saharan Africa and Europe: Current Landscape and Potential for Future Research. Front. Public Health.

[34] Silver L., Johnson C. (2018). Majorities in Sub-Saharan Africa Own Mobile Phones, but Smartphone Adoption Is Modest. Pew Research Center’s Global Attitudes Project.

[35] Olanrewaju B. (2021). Youth Driving Smartphone Penetration in Africa to Increase by 30% in 10 Years—Report. Business Remarks.

[36] Makri A. (2019). Bridging the Digital Divide in Health Care. Lancet Digit. Health.

[37] Houngbonon G.V., Le Quentrec E., Rubrichi S. (2021). Access to Electricity and Digital Inclusion: Evidence from Mobile Call Detail Records. Hum. Soc. Sci Commun..

[38] Carrouel F., Bourgeois D., Clément C., Tardivo D., Martinon P., Guiral S., Lan R., Viennot S., Dussart C., Fraticelli L. (2022). Oral-Hygiene-Related Mobile Apps in the French App Stores: Assessment of Functionality and Quality. Int. J. Environ. Res. Public Health.

[39] Tiffany B., Blasi P., Catz S.L., McClure J.B. (2018). Mobile Apps for Oral Health Promotion: Content Review and Heuristic Usability Analysis. JMIR mHealth uHealth.

[40] Sharif M.O., Alkadhimi A. (2019). Patient Focused Oral Hygiene Apps: An Assessment of Quality (Using MARS) and Knowledge Content. Br. Dent. J..

[41] Parker K., Bharmal R.V., Sharif M.O. (2019). The Availability and Characteristics of Patient-Focused Oral Hygiene Apps. Br. Dent. J..

[42] Chen R., Santo K., Wong G., Sohn W., Spallek H., Chow C., Irving M. (2021). Mobile Apps for Dental Caries Prevention: Systematic Search and Quality Evaluation. JMIR mHealth uHealth.

[43] Eidenhardt Z., Ritsert A., Shankar-Subramanian S., Ebel S., Margraf-Stiksrud J., Deinzer R. (2021). Tooth Brushing Performance in Adolescents as Compared to the Best-Practice Demonstrated in Group Prophylaxis Programs: An Observational Study. BMC Oral Health.

[44] (2000). Oral Health in America: A Report of the Surgeon General. J. Calif. Dent. Assoc..

[45] de Chantal P.-L., Chagnon A., Cardinal M., Faieta J., Guertin A. (2022). Evidence of User-Expert Gaps in Health App Ratings and Implications for Practice. Front. Digit. Health.

[46] Martinon P., Saliasi I., Bourgeois D., Smentek C., Dussart C., Fraticelli L., Carrouel F. (2022). Nutrition-Related Mobile Apps in the French App Stores: Assessment of Functionality and Quality. JMIR mHealth uHealth.

[47] Schumer H., Amadi C., Joshi A. (2018). Evaluating the Dietary and Nutritional Apps in the Google Play Store. Healthc Inf. Res..

[48] Fijačko N., Gosak L., Cilar L., Novšak A., Creber R.M., Skok P., Štiglic G. (2020). The Effects of Gamification and Oral Self-Care on Oral Hygiene in Children: Systematic Search in App Stores and Evaluation of Apps. JMIR mHealth uHealth.

[49] Stoyanov S.R., Hides L., Kavanagh D.J., Wilson H. (2016). Development and Validation of the User Version of the Mobile Application Rating Scale (UMARS). JMIR mHealth uHealth.

[50] Farhadifard H., Soheilifar S., Farhadian M., Kokabi H., Bakhshaei A. (2020). Orthodontic Patients’ Oral Hygiene Compliance by Utilizing a Smartphone Application (Brush DJ): A Randomized Clinical Trial. BDJ Open.

[51] Underwood B., Birdsall J., Kay E. (2015). The Use of a Mobile App to Motivate Evidence-Based Oral Hygiene Behaviour. Br. Dent. J..

[52] Zahid T., Alyafi R., Bantan N., Alzahrani R., Elfirt E. (2020). Comparison of Effectiveness of Mobile App versus Conventional Educational Lectures on Oral Hygiene Knowledge and Behavior of High School Students in Saudi Arabia. Patient Prefer. Adherence.

[53] Becker S., Miron-Shatz T., Schumacher N., Krocza J., Diamantidis C., Albrecht U.-V. (2014). MHealth 2.0: Experiences, Possibilities, and Perspectives. JMIR mHealth uHealth.

[54] DiFilippo K.N., Huang W., Chapman-Novakofski K.M. (2017). A New Tool for Nutrition App Quality Evaluation (AQEL): Development, Validation, and Reliability Testing. JMIR mHealth uHealth.

