# Supplementary material for: Evaluation of Oral Hygiene-Related Mobile Apps for Children in Sub-Saharan Africa
Source: Int J Environ Res Public Health. 2022 Oct 1;19(19):12565. doi: 10.3390/ijerph191912565 (PMC9565087; doi:10.3390/ijerph191912565)
Supplement: Supplementary file 1 [file ijerph-19-12565-s001.zip › ijerph-1887907-supplementary.pdf]

Table S1. STROBE Statement—checklist of items that should be included in reports of observational studies.

	Item No	Recommendation	Page No
**Title and abstract**	1	(*a*) Indicate the study's design with a commonly used term in the title or the abstract	1
		(*b*) Provide in the abstract an informative and balanced summary of what was done and what was found	1
**Introduction**			
Background/rationale	2	Explain the scientific background and rationale for the investigation being reported	1-2
Objectives	3	State specific objectives, including any prespecified hypotheses	2
**Methods**			
Study design	4	Present key elements of study design early in the paper	2
Setting	5	Describe the setting, locations, and relevant dates, including periods of recruitment, exposure, follow-up, and data collection	2-3
Participants	6	(*a*) *Cohort study*—Give the eligibility criteria, and the sources and methods of selection of participants. Describe methods of follow-up<br>*Case-control study*—Give the eligibility criteria, and the sources and methods of case ascertainment and control selection. Give the rationale for the choice of cases and controls<br>*Cross-sectional study*—Give the eligibility criteria, and the sources and methods of selection of participants	3
		(*b*) *Cohort study*—For matched studies, give matching criteria and number of exposed and unexposed<br>*Case-control study*—For matched studies, give matching criteria and the number of controls per case	NA
Variables	7	Clearly define all outcomes, exposures, predictors, potential confounders, and effect modifiers. Give diagnostic criteria, if applicable	3
Data sources/ measurement	8*	For each variable of interest, give sources of data and details of methods of assessment (measurement). Describe comparability of assessment methods if there is more than one group	*3-4*
Bias	9	Describe any efforts to address potential sources of bias	*3-4*
Study size	10	Explain how the study size was arrived at	3
Quantitative variables	11	Explain how quantitative variables were handled in the analyses. If applicable, describe which groupings were chosen and why	3-4
Statistical methods	12	(*a*) Describe all statistical methods, including those used to control for confounding	4
		(*b*) Describe any methods used to examine subgroups and interactions	3-4
		(*c*) Explain how missing data were addressed	NA
		(*d*) *Cohort study*—If applicable, explain how loss to follow-up was addressed<br>*Case-control study*—If applicable, explain how matching of cases and controls was addressed<br>*Cross-sectional study*—If applicable, describe analytical methods taking account of sampling strategy	NA
		(*e*) Describe any sensitivity analyses	NA

Continued on next page

**Results**			
Participants	13*	(a) Report numbers of individuals at each stage of study—eg numbers potentially eligible, examined for eligibility, confirmed eligible, included in the study, completing follow-up, and analysed	NA
		(b) Give reasons for non-participation at each stage	NA
		(c) Consider use of a flow diagram	NA
Descriptive data	14*	(a) Give characteristics of study participants (eg demographic, clinical, social) and information on exposures and potential confounders	S2
		(b) Indicate number of participants with missing data for each variable of interest	NA
		(c) *Cohort study*—Summarise follow-up time (eg, average and total amount)	NA
Outcome data	15*	*Cohort study*—Report numbers of outcome events or summary measures over time	*NA*
		Case-control study—Report numbers in each exposure category, or summary measures of exposure	*NA*
		Cross-sectional study—Report numbers of outcome events or summary measures	*NA*
Main results	16	(*a*) Give unadjusted estimates and, if applicable, confounder-adjusted estimates and their precision (eg, 95% confidence interval). Make clear which confounders were adjusted for and why they were included	4-10
		(*b*) Report category boundaries when continuous variables were categorized	NA
		(*c*) If relevant, consider translating estimates of relative risk into absolute risk for a meaningful time period	NA
Other analyses	17	Report other analyses done—eg analyses of subgroups and interactions, and sensitivity analyses	NA
**Discussion**			
Key results	18	Summarise key results with reference to study objectives	11-13
Limitations	19	Discuss limitations of the study, taking into account sources of potential bias or imprecision. Discuss both direction and magnitude of any potential bias	13
Interpretation	20	Give a cautious overall interpretation of results considering objectives, limitations, multiplicity of analyses, results from similar studies, and other relevant evidence	11-13
Generalisability	21	Discuss the generalisability (external validity) of the study results	11-13
**Other information**			
Funding	22	Give the source of funding and the role of the funders for the present study and, if applicable, for the original study on which the present article is based	NA

*Give information separately for cases and controls in case-control studies and, if applicable, for exposed and unexposed groups in cohort and cross-sectional studies.

Note: An Explanation and Elaboration article discusses each checklist item and gives methodological background and published examples of transparent reporting. The STROBE checklist is best used in conjunction with this article (freely available on the Web sites of PLoS Medicine at http://www.plosmedicine.org/, Annals of Internal Medicine at http://www.annals.org/, and Epidemiology at http://www.epidem.com/). Information on the STROBE Initiative is available at www.strobe-statement.org.

Table S2. Characteristics of the raters, the hardware and the software used.

	**Rater 1**	**Rater 2**	**Rater 3**	**Rater 4**	**Rater 5**	**Rater 6**	**Rater 7**	**Rater 8**	**Rater 9**	**Rater 10**
**Year of experience as dentist**	7	22	15	2	17	14	2	16	15	41
**Affiliation**	Hospital and University	Hospital and University	University	Hospital	Liberal and Hospital	University	Hospital	Liberal and Hospital	Hospital	Liberal and Hospital
**Scope of activity**	Public health	Public health and Pedodontist	Public health	General practitioner	Prosthetist	Public health	General practitioner	Periodontist	Prosthetist	Periodontist – Public health
**Hardware (phone)**	Infinix Note 10	Samsung Galaxy S21	Iphone 11	Iphone 11	Hawaii	Samsung A22	Samsung A12	Iphone 11	Iphone 11	Iphone 12 mini
**Software (iOS or Android)**	Android	Android	iOS	iOS	Android	Android	Android	iOS	iOS	iOS

Table S3. Developer, Scare rating and Paid content of the 15 oral-health-related mobile applications, for children, included in the study.

App name	Developer	Rating in the iOS app store (Nb of raters)	Rating in the Android app store (Nb of raters /Nb of dowloads)	Paid Content
Apprendre avec Ben le Koala	Signes de sens	NA (NA)	NA (NA/>100 000)	Free App
Bonne nuit Caillou	Budge Studios	NA (NA)	NA (NA/>10M)	Free with in-app purchases
Brosse à dents	BABYBUS CO., LTD	NA (NA)	4.3 (24855/>5M)	Free with in-app purchases
Brush DJ	Ben Underwood	3 (1)	NA (NA/>100 000)	Free App
Brush jam	Saj Arora	3 (1)	NA (NA/>100)	Free App
BrushYourTeeth	puripuri	NA (NA)	NA (NA/>10 000)	Free App
Brushing Hero	LITALICO lnc.	NA (NA)	NA (NA/>100 000)	Free with in-app purchases
Brushing time	Kwangyoung Jung	NA (NA)	NA (NA/>100)	Free App
Chomper Chums	United Concordia Dental	NA (NA)	NA (NA/>100 000)	Free App
De belles dents	Erwan Peres	NA (NA)	NA (NA/>100)	Free App
Disney magic Timer by Oral-B	Disney	NA (NA)	NA (NA/>5M)	Free App
Happy kids Timer – Matin	Kids Smart Zone - Best apps and games for your kids	NA (NA)	4.6 (20133/>1M)	Free with in-app purchases
Mimizaur se brosse les dents	Shorokh Natalya	NA (NA)	NA (NA/>50 000)	Free with in-app purchases
Mon Raccoon	Pierre Fabre Médicament	NA (NA)	NA (NA/>5 000)	Free App
Pokémon Smile	The Pokemon Company	1 (1)	NA (NA/1M)	Free App

Table S4. Brief description of the 15 oral-health-related mobile applications, for children, included in the study.

App name	Brief description of app
Apprendre avec Ben le Koala	This application aims to teach children, thanks to the main character Ben Koala, the daily gestures and autonomy. In this application, there are videos to learn while having fun to brush the teeth...
Bonne nuit Caillou	This application aims to teach children, thanks to the main character Caillou, the daily gestures of bedtime and autonomy. In this application, there are videos to learn while having fun to brush the teeth...
Brosse à dents	This application aims to teach children how to brush their teeth thanks to the character called Baby Brossadent.
Brush DJ	This application is a toothbrush timer app that plays 2 minutes of music to make toothbrushing for an effective length of time.
Brush jam	This application is a toothbrush timer app that plays 2 minutes of music to make toothbrushing for an effective length of time.
BrushYourTeeth	This application is a toothbrush timer app that plays 2 minutes of music to make toothbrushing for an effective length of time. It also indicates where brush.
Brushing Hero	This application makes tooth brushing fun for kids by transforming them into heroes wearing an iron war helmet. When children brush their teeth, they attack the monsters and can move to the next stage of the game.
Brushing time	This application helps to brush for 2 minutes and track brushing history.
Chomper Chums	The application aims to help children to learn proper brushing techniques and to establish long lasting and healthy oral wellness habits.
De belles dents	This app is a 2- or 3-minutes timer that ensures the recommended minimum tooth brushing time is observed.
Disney magic Timer by Oral-B	This application aims to increase tooth brushing time in children by using Disney characters and fun games.
Happy kids Timer – Matin	This application is a timer that helps children to do all the daily tasks of the morning or evening including brushing their teeth. Children earn stars at each step.
Mimizaur se brosse les dents	This application aims to learn how to brush your teeth through different cartoons whose main character is Mimizaur.
Mon Raccoon	This application aims to develop oral and food hygiene skills in children.
Pokémon Smile	This application makes tooth brushing fun for kids by transforming them into Pokémon. When children brush their teeth, they help their favorite Pokémon to fight against bacteria.

Table S5. Targets of the 15 oral-health-related mobile applications, for children, included in the study.

**Focus: what the app targets**	Increase Happiness/Well-being	Mindfulness/Meditation/Relaxation	Anxiety/Stress	Behavior Change	Goal Setting	Entertainment	Relationships	Physical health
Apprendre avec Ben le Koala	X			X	X	X	X	
Bonne nuit Caillou	X			X	X	X	X	
Brosse à dents	X			X	X	X	X	
Brush DJ	X			X	X	X		
Brush jam	X			X	X	X		
BrushYourTeeth				X	X			
Brushing Hero	X			X	X	X	X	
Brushing time				X	X			
Chomper Chums	X			X	X	X	X	
De belles dents				X	X			
Disney magic Timer by Oral-B	X			X	X	X	X	
Happy kids Timer – Matin	X			X	X	X	X	
Mimizaur se brosse les dents	X			X	X	X	X	
Mon Raccoon	X			X	X	X	X	
Pokémon Smile	X			X	X	X	X	

Table S6. Theoretical background and strategies of the 15 oral-health-related mobile applications, for children, included in the study.

**Theoretical background/Strategies**	Assessment	Feedback	Information/Education	Monitoring/Tracking	Goal Setting	Advice /Tips /Strategies /Skills training	CBT - Behavioral (positive events)	CBT – Cognitive (thought challenging)	ACT - Acceptance commitment therapy	Mindfulness/Meditation	Relaxation	Gratitude	Strengths based
Apprendre avec Ben le Koala			X		X	X	X						
Bonne nuit Caillou			X		X	X	X					X	
Brosse à dents			X		X	X	X					X	
Brush DJ			X		X		X						
Brush jam			X		X		X						
BrushYourTeeth			X		X		X						
Brushing Hero			X		X	X	X					X	
Brushing time			X	X	X	X	X						
Chomper Chums			X	X	X	X	X					X	
De belles dents			X		X		X						
Disney magic Timer by Oral-B			X	X	X	X	X					X	
Happy kids Timer – Matin			X	X	X	X	X					X	
Mimizaur se brosse les dents			X				X						
Mon Raccoon			X	X	X	X	X					X	
Pokémon Smile			X	X	X	X	X					X	

Table S7. Affiliations of the 15 oral health related mobile applications, for children, included in the study.

	Unknown	Commercial	Government	Non-governmental association
Apprendre avec Ben le Koala	X			
Bonne nuit Caillou		X		
Brosse à dents		X		
Brush DJ	X			
Brush jam	X			
BrushYourTeeth	X			
Brushing Hero		X		
Brushing time	X			
Chomper Chums	X			
De belles dents	X			
Disney magic Timer by Oral-B		X		
Happy kids Timer – Matin	X			
Mimizaur se brosse les dents		X		
Mon Raccoon		X		
Pokémon Smile		X		

Table S8. Age group of the 15 oral-health-related mobile applications, for children, included in the study.

**Age group**	Children (under 12)	Adolescents (13-17)	Young Adults (18-25)	Adults
Apprendre avec Ben le Koala	X			
Bonne nuit Caillou	X			
Brosse à dents	X			
Brush DJ	X	X	X	X
Brush jam	X	X	X	X
BrushYourTeeth	X	X	X	X
Brushing Hero	X			
Brushing time	X	X	X	X
Chomper Chums	X			
De belles dents	X	X	X	X
Disney magic Timer by Oral-B	X			
Happy kids Timer – Matin	X			
Mimizaur se brosse les dents	X			
Mon Raccoon	X			
Pokémon Smile	X			

Table S9. Technical aspects of the 15 oral-health-related mobile applications, for children, included in the study.

**Technical aspects of app**	Allows sharing (Facebook, Twitter, etc.)	Has an app community	Allows password-protection	Requires login	Sends reminders	Needs web access to function
Apprendre avec Ben le Koala			X			X
Bonne nuit Caillou						
Brosse à dents						
Brush DJ					X	
Brush jam	X					X
BrushYourTeeth						
Brushing Hero						
Brushing time						
Chomper Chums						
De belles dents						
Disney magic Timer by Oral-B						
Happy kids Timer – Matin						
Mimizaur se brosse les dents						
Mon Raccoon						
Pokémon Smile						

Table S10. Mobile App Rating Scale-French (MARS-F) scoring by section for the 15 oral-health-related mobile applications, for children, included in the study.

App Name	Data	Section A	Section B	Section C	Section D	Section ABCD	Section E	Section F
Apprendre avec Ben le Koala	Mean	2.96	3.70	3.40	3.25	3.33	2.58	3.07
	SD	0.87	0.60	0.62	0.56	0.51	0.87	0.87
Bonne nuit Caillou	Mean	3.74	3.95	4.07	3.82	3.89	3.25	3.75
	SD	0.95	0.74	0.81	0.80	0.74	0.97	0.84
Brosse à dents	Mean	2.92	3.65	3.53	3.05	3.29	2.33	3.02
	SD	0.80	0.81	0.39	0.66	0.55	0.90	1.08
Brush DJ	Mean	2.56	3.50	3.03	3.13	3.06	1.98	2.75
	SD	0.82	0.74	0.90	0.29	0.52	0.79	0.79
Brush jam	Mean	3.32	3.35	3.27	3.03	3.24	2.30	2.78
	SD	0.86	0.59	0.83	0.44	0.59	0.93	0.65
BrushYourTeeth	Mean	3.04	3.28	3.30	2.90	3.13	2.35	2.58
	SD	1.04	1.24	0.81	0.74	0.86	1.11	1.04
Brushing Hero	Mean	2.34	3.40	2.77	2.58	2.77	1.60	2.42
	SD	0.58	1.01	0.90	0.44	0.53	0.65	0.85
Brushing time	Mean	2.00	2.70	2.03	2.50	2.31	1.50	1.95
	SD	0.93	1.05	0.82	0.35	0.61	0.68	0.88
Chomper Chums	Mean	3.60	3.70	3.63	3.23	3.54	2.58	3.27
	SD	0.77	0.54	0.46	0.51	0.54	0.98	0.62
De belles dents	Mean	1.94	3.25	2.50	2.52	2.55	1.68	2.23
	SD	0.67	0.91	0.79	0.51	0.55	0.69	0.69
Disney magic Timer by Oral-B	Mean	3.46	3.50	3.83	3.28	3.52	2.75	3.25
	SD	0.72	0.75	0.61	0.50	0.59	1.08	1.10
Happy kids Timer – Matin	Mean	3.24	3.50	3.43	3.22	3.35	2.25	2.52
	SD	0.74	0.87	0.75	0.65	0.69	1.08	0.68
Mimizaur se brosse les dents	Mean	3.24	3.60	3.37	3.13	3.34	2.73	3.27
	SD	0.80	0.54	0.48	0.42	0.47	1.04	0.69
Mon Raccoon	Mean	3.84	3.70	3.70	3.27	3.63	2.88	3.60
	SD	0.95	1.14	1.02	0.88	0.95	1.21	1.14
Pokémon Smile	Mean	3.52	3.50	3.63	3.10	3.44	2.68	3.32
	SD	0.89	0.94	0.76	0.83	0.82	1.08	1.07

Table S11. Mobile App Rating Scale-French (MARS-F) scoring by items the 15 oral health related mobile applications, for children, included in the study.

App Name	Data	Section A					Section B				Section C			Section D						
		Item 1	Item 2	Item 3	Item 4	Item 5	Item 6	Item 7	Item 8	Item 9	Item 10	Item 11	Item 12	Item 13	Item 14	Item 15	Item 16	Item 17	Item 18	Item 19
Apprendre avec Ben le Koala	Mean	3.40	3.40	2.40	2.30	3.30	3.40	3.60	3.90	3.90	3.70	3.20	3.30	3.80	3.30	3.20	3.50	3.80	1.90	NA
	SD	0.70	0.97	1.43	1.70	0.95	0.97	0.70	0.88	0.57	0.67	0.79	0.67	0.92	0.48	1.03	0.71	0.79	0.74	NA
Bonne nuit Caillou	Mean	4.20	4.00	2.90	3.40	4.20	3.80	3.70	4.10	4.20	4.00	4.10	4.10	4.10	4.00	3.80	4.10	4.40	2.50	NA
	SD	0.92	1.25	1.37	0.97	0.79	0.79	1.06	0.74	0.79	0.94	0.74	0.88	1.10	0.82	1.14	0.88	0.70	1.35	NA
Brosse à dents	Mean	2.90	3.30	2.00	2.70	3.70	3.80	3.60	3.70	3.50	4.00	3.30	3.30	3.30	3.20	3.30	2.90	3.30	2.30	NA
	SD	0.99	1.25	0.94	1.06	0.82	0.79	1.07	1.06	0.97	0.47	0.67	0.48	1.25	0.63	0.82	0.88	1.06	1.25	NA
Brush DJ	Mean	2.30	2.70	2.70	2.50	2.60	3.90	3.40	3.60	3.10	3.50	2.90	2.70	3.50	2.90	3.20	3.30	3.90	2.00	NA
	SD	0.67	0.95	1.25	1.27	1.17	0.74	1.07	0.70	1.10	0.97	0.88	1.06	0.97	0.32	0.63	0.48	0.57	0.94	NA
Brush jam	Mean	3.60	3.50	3.20	2.70	3.60	3.40	3.20	3.50	3.30	3.50	3.20	3.10	3.10	3.10	3.20	3.10	3.80	1.90	NA
	SD	1.07	0.85	1.03	1.06	0.84	0.52	1.03	0.71	0.67	0.71	1.23	0.99	1.37	0.32	0.63	0.32	0.63	0.74	NA
BrushYourTeeth	Mean	3.70	3.60	2.20	2.70	3.00	3.40	3.40	3.20	3.10	3.10	3.50	3.30	3.00	3.10	2.90	2.90	3.70	1.80	NA
	SD	1.34	1.35	1.03	0.95	1.41	1.17	1.51	1.32	1.29	0.99	0.85	0.82	0.94	0.88	1.29	0.88	0.95	0.79	NA
Brushing Hero	Mean	2.30	2.20	2.40	1.80	3.00	3.40	3.50	3.30	3.40	3.40	2.60	2.30	2.80	3.00	2.70	2.80	2.80	1.40	NA
	SD	0.95	0.63	0.84	0.79	0.47	0.84	1.35	1.25	1.35	1.17	0.84	0.95	0.92	0.47	0.82	0.79	1.14	0.52	NA
Brushing time	Mean	2.30	2.10	1.50	1.60	2.50	2.90	2.60	2.80	2.50	1.90	2.00	2.20	2.20	3.00	2.60	2.60	3.10	1.50	NA
	SD	1.25	1.29	0.85	0.84	1.18	1.20	1.35	1.32	1.27	0.99	0.94	0.92	0.79	0.00	0.52	0.97	0.88	0.71	NA
Chomper Chums	Mean	4.00	4.00	3.30	3.20	3.50	3.60	3.80	3.90	3.50	3.70	3.70	3.50	3.80	3.50	3.20	3.30	3.40	2.20	NA
	SD	0.94	0.94	0.67	1.03	0.71	0.52	0.63	0.57	0.97	0.48	0.48	0.53	0.79	0.53	0.63	0.67	0.70	1.03	NA
De belles dents	Mean	1.90	2.30	1.80	1.50	2.20	3.40	3.30	3.30	3.00	3.10	2.30	2.10	2.40	3.00	2.50	2.40	2.80	2.00	NA
	SD	0.88	0.95	0.79	0.71	0.92	1.17	1.34	1.25	1.05	1.10	0.82	0.74	0.97	0.94	0.71	0.70	0.92	0.82	NA
Disney magic Timer by Oral-B	Mean	3.90	3.80	3.30	2.70	3.60	3.70	3.20	3.40	3.70	3.60	4.00	3.90	3.70	3.30	3.30	3.50	3.60	2.30	NA
	SD	0.88	1.03	0.95	0.95	0.70	0.67	0.79	0.97	0.82	0.84	0.82	0.57	0.48	0.67	0.67	0.85	0.84	0.95	NA
Happy kids Timer – Matin	Mean	3.30	3.30	2.90	3.30	3.40	3.80	3.40	3.60	3.20	3.60	3.40	3.30	3.50	3.00	3.50	3.50	3.70	2.10	NA
	SD	1.06	1.16	1.20	0.67	0.84	0.79	1.35	1.17	0.92	0.97	0.70	0.82	1.08	0.82	0.71	0.97	0.67	0.88	NA
Mimizaur se brosse les dents	Mean	3.60	3.70	2.60	2.50	3.80	3.70	3.20	3.70	3.80	3.40	3.30	3.40	3.60	3.00	3.40	3.30	3.60	1.90	NA
	SD	1.07	1.06	0.84	1.18	1.03	0.67	1.03	0.67	0.79	0.52	0.48	0.52	0.70	0.47	0.52	0.48	0.70	0.74	NA
Mon Raccoon	Mean	3.80	4.00	3.80	3.50	4.10	3.60	3.50	3.90	3.80	3.80	3.60	3.70	3.60	3.20	3.50	3.30	3.50	2.50	NA
	SD	1.14	1.05	0.92	1.18	0.74	1.26	1.51	0.99	1.03	1.03	1.17	0.95	0.97	1.03	0.97	0.95	0.97	0.97	NA
Pokémon Smile	Mean	3.80	3.80	3.30	3.30	3.40	3.50	3.50	3.50	3.50	3.60	3.70	3.60	3.50	2.80	3.60	3.20	3.60	1.90	NA
	SD	1.14	1.03	0.95	0.95	0.84	1.08	0.97	1.08	0.85	0.70	0.95	0.84	0.97	1.14	0.70	1.14	1.17	0.88	NA

App Name	Data	Section E				Mobile app specificities					
		Item 20	Item 21	Item 22	Item 23	Awareness	Knowledge	Attitudes	Intention to change	Help seeking	Behavior change
Apprendre avec Ben le Koala	Mean	2.80	2.60	1.80	3.10	3.10	3.10	3.00	2.90	3.20	3.10
	SD	1.03	1.17	1.03	0.88	1.10	0.88	0.94	0.99	1.03	0.88
Bonne nuit Caillou	Mean	3.60	3.30	2.40	3.70	3.80	3.70	3.80	3.80	3.60	3.80
	SD	1.17	1.16	1.65	0.82	0.92	0.95	0.79	0.79	0.97	0.79
Brosse à dents	Mean	2.60	2.40	1.40	2.90	3.30	3.10	2.80	3.00	2.70	3.20
	SD	1.07	1.17	0.84	1.10	1.16	1.29	1.14	1.15	1.06	1.14
Brush DJ	Mean	2.30	2.00	1.20	2.40	2.60	2.80	2.80	2.70	2.60	3.00
	SD	1.25	1.15	0.63	0.70	0.97	1.14	0.92	1.06	0.70	0.82
Brush jam	Mean	2.60	2.30	1.60	2.70	2.70	2.90	2.90	2.80	2.70	2.70
	SD	1.07	1.34	0.97	0.95	0.67	0.74	0.74	0.63	0.67	0.67
BrushYourTeeth	Mean	2.40	2.70	1.60	2.70	2.60	2.40	2.60	2.80	2.50	2.60
	SD	1.26	1.34	0.97	1.16	1.07	0.97	1.07	1.23	0.97	1.17
Brushing Hero	Mean	1.50	1.60	1.00	2.30	2.40	2.50	2.40	2.30	2.40	2.50
	SD	0.71	1.07	0.00	1.16	0.84	0.85	0.84	0.82	0.84	1.08
Brushing time	Mean	1.50	1.50	1.20	1.80	2.00	1.80	1.80	2.00	2.10	2.00
	SD	0.85	0.85	0.63	0.79	1.05	0.79	0.79	1.05	0.88	0.94
Chomper Chums	Mean	2.80	2.70	1.60	3.20	3.40	3.40	3.20	3.20	3.00	3.40
	SD	1.03	1.49	0.97	1.03	0.84	0.70	0.63	0.63	0.67	0.70
De belles dents	Mean	1.60	1.70	1.40	2.00	2.20	2.50	2.30	2.30	1.70	2.40
	SD	0.70	0.95	0.84	0.94	0.79	0.85	0.67	0.67	0.82	0.97
Disney magic Timer by Oral-B	Mean	2.80	3.00	1.80	3.40	3.30	3.10	3.40	3.30	3.10	3.30
	SD	1.03	1.56	1.03	1.17	1.16	1.10	1.26	1.25	0.99	1.16
Happy kids Timer – Matin	Mean	2.30	2.20	1.80	2.70	2.70	2.20	2.70	2.80	2.30	2.40
	SD	1.25	1.23	1.03	1.06	0.82	0.79	1.06	0.79	0.67	0.84
Mimizaur se brosse les dents	Mean	3.00	2.80	2.00	3.10	3.30	3.20	3.20	3.40	3.20	3.30
	SD	1.25	1.23	1.41	0.99	0.67	0.79	0.63	0.84	0.79	0.67
Mon Raccoon	Mean	3.00	2.90	2.00	3.60	3.60	3.60	3.60	3.60	3.60	3.60
	SD	1.33	1.45	1.41	1.26	1.17	1.17	1.17	1.17	1.07	1.17
Pokémon Smile	Mean	2.50	2.60	2.00	3.60	3.30	3.30	3.40	3.40	3.10	3.40
	SD	1.27	1.35	1.41	0.84	1.16	1.06	1.26	1.26	0.74	1.26
